# Preventive effects of low-dose aspirin on colorectal adenoma growth in patients with familial adenomatous polyposis: double-blind, randomized clinical trial

**DOI:** 10.1002/cam4.46

**Published:** 2013-02-03

**Authors:** Hideki Ishikawa, Keiji Wakabayashi, Sadao Suzuki, Michihiro Mutoh, Keiji Hirata, Tomiyo Nakamura, Ikuko Takeyama, Atsuko Kawano, Nobuhisa Gondo, Takashi Abe, Shinkan Tokudome, Chiho Goto, Nariaki Matsuura, Toshiyuki Sakai

**Affiliations:** 1 Department of Molecular-Targeting Cancer Prevention, Graduate School of Medical Science, Kyoto Prefectural University of MedicineKyoto, Japan; 2 Graduate School of Integrated Pharmaceutical and Nutritional Sciences, University of ShizuokaShizuoka, Japan; 3 Department of Public Health, Nagoya City University Graduate School of Medical SciencesNagoya, Japan; 4 Division of Cancer Preventive Research, National Cancer Center Research InstituteTokyo, Japan; 5 Department of Surgery, Fukuoka Sanno HospitalFukuoka, Japan; 6 Department of Molecular Pathology, Osaka University Graduate School of Medicine and Health ScienceOsaka, Japan; 7 Department of Clinical NutritionFaculty of Comprehensive Rehabilitation, Osaka Prefecture UniversityOsaka, Japan; 8 Department of Hygiene and Public Health, Osaka Medical CollegeOsaka, Japan; 9 Department of Clinical Genetics, Hyogo College of MedicineHyogo, Japan; 10 Department of Gastroenterology, Takarazuka Municipal HospitalHyogo, Japan; 11 National Institute of Health and NutritionTokyo, Japan; 12 Department of Health and NutritionSchool of Health and Human Life, Nagoya Bunri UniversityAichi, Japan

**Keywords:** Adenoma, chemoprevention, colorectum, FAP, low-dose aspirin

## Abstract

There are several reports of clinical trials of aspirin in sporadic colon cancer. However, only one double-blind trial of aspirin in patients with familial adenomatous polyposis (FAP) has been reported to date. This double-blind, randomized, placebo-controlled clinical trial was therefore performed to evaluate the influence of low-dose aspirin enteric-coated tablets (100 mg/day for 6–10 months) in 34 subjects with FAP (17 each in the aspirin and placebo groups). The increase in mean diameter of colorectal polyps tended to be greater in the placebo group compared with the aspirin group, which showed a response ratio, that is, aspirin response rate (number of subjects with reduced polyps/total)/placebo response rate (number of subjects with reduced polyps/total), of 2.33 (95% confidence interval: 0.72–7.55). Subgroup analysis revealed that the number of subjects with a mean baseline polyp diameter of ≤2 mm, and the diameter and number of polyps after intervention showed a significant reduction in the aspirin group. Adverse effects of aspirin, such as anastomotic ulcer, aphtha in the large intestine, and progression of anemia, occurred in three subjects. Moreover, none of the subjects developed colorectal cancer. The results thus indicated a potential for aspirin to reduce colorectal adenoma development in patients with FAP, but careful follow-up is needed to avoid or rapidly counter severe adverse effects.

## Introduction

Familial adenomatous polyposis (FAP) is a very rare autosomal dominant inherited disorder, characterized by the occurrence of many adenomas in the large intestine. It has been reported that the polyps in the colorectum progress to adenocarcinoma in half of the patient population by the age of 40 years [1].

Hitherto, complete surgical removal of the colorectum was the only preventive method against colorectal cancer development in FAP patients. This intervention, however, has caused frequent diarrhea, which led to a significant decrease in patients’ quality of life. Therefore, research on chemoprevention has been conducted in the hope that it may be able to delay surgery or, in patients with a smaller number of polyps, help avoid surgery by combining it with endoscopic resection [2]. Among candidate substances, sulindac, a nonsteroidal anti-inflammatory drug (NSAID), has been studied frequently in a clinical setting [3–5]. Giardiello et al. reported that sulindac reduced the number and size of colorectal adenomas in a double-blind randomized trial [3]. Although many articles indicate that sulindac may safely inhibit colorectal adenoma, there is just one article that reported the possibility that long-term administration of sulindac may cause adverse effects such as gastric ulcer and anemia [4]. Although hopes have focused on cyclooxygenase-2 (COX-2)-selective inhibitors, including celecoxib and rofecoxib, causing little damage to the gastric mucosa [6,7], recent studies have revealed that they may evoke cardiotoxicity [8,9].

In the recent past, a great deal of experience has been gained with long-term use of aspirin tablets for antiplatelet therapy for cardiovascular diseases around the world. This treatment is relatively well tolerated and clinical trials have demonstrated protective effects of aspirin against sporadic colorectal adenomas as well as colorectal cancers [11–13]. However, to our knowledge, only one clinical trial of aspirin in patients with FAP has been reported to date [14]. Here, we report efficacy and safety of low-dose aspirin enteric-coated tablets for suppression of intestinal polyp development in FAP patients, with special attention to control of adverse effects.

## Materials and Methods

### Trial methodology

In this double-blind, randomized, placebo-controlled trial using low-dose aspirin enteric-coated tablets, the subjects received either 100 mg/day aspirin or placebo for 6–10 months. The duration of 6–10 months was selected because the periodic colonoscopy for FAP patients was 6–10 months. Random assignment was performed in each case by an investigator using a computer-aided system on the Medical Research Support Web site (Kyoto, Japan). This web site was available only to the trial participants. We used a minimization algorithm, one of the dynamic allocation methods for clinical trials, to achieve a balance between treatment groups with respect to four stratification variables: institution, sex, age (<30 or ≥30 years), and history of colorectal surgery. Enrollment of the subjects started in June 2007 and the trial was completed in October 2009. Osaka Central Hospital and the University of Occupational and Environmental Health Hospital participated in this trial, for which an Ethics Monitoring Committee was established. A system to ensure continuous follow-up of adverse effects was also installed. Each of the two trial sites obtained approval from its own ethics committee for the conduct of the trial.

### Trial population

The trial population was patients with FAP, defined as the presence of ≥100 adenomas in the large intestine, or a germline mutation in the adenomatous polyposis coli (*APC*) gene. All the subjects participating in the trial had an intact rectum or a residual rectum at least 2 cm in length, were aged ≥16 and ≤70 years, and were Japanese. As FAP patients with preserved colonic mucosa had received colonoscopy regularly and already had polyps 5 mm or larger removed endoscopically, there was no polyp of 5 mm or larger at the time of recruitment.

The following were excluded from the trial: (1) patients with active cancer at the time of entry, (2) those currently taking an antithrombotic or anticoagulant, (3) individuals with a history of stroke or gastric/duodenal ulcers (except for those with scars healed as confirmed after successful eradiation of *Helicobacter pylori*), (4) those with inflammatory enteritis, hemorrhagic diverticulitis, or bleeding tendency, (5) those with a platelet count of ≤100,000/mm^3^, those with abnormal prothrombin time, (6) people with known allergy to aspirin, (7) those who were pregnant or planned to become pregnant during the trial period, and (8) those taking an NSAID for pain relief more than three times weekly. Subjects who had previously been treated with sulindac were allowed to participate in the trial with the limitation that the drug had been discontinued more than 6 months prior to the start of the trial.

Consent interviews were individually performed and informed consent regarding participation in the trial was obtained from each patient.

### Investigational drug

Low-dose aspirin enteric-coated tablets (100 mg per tablet) and placebo counterparts provided by Bayer in Germany were imported to Japan. This trial was financed by research funding by the Ministry of Health, Labour and Welfare. We signed an agreement to assure that we had no conflict of interest for Bayer. The investigational drugs were placed in blister packages (calendar sheets of 31 tablets), both sides of which were aluminum laminated.

### Study questionnaire

At the trial entry, height, body weight, medical history, and use of NSAIDs were investigated for each patient through a questionnaire. In addition, data regarding everyday meals were collected using a self-administered food-frequency questionnaire developed by the Department of Health and Nutrition, Nagoya-bunri University, Aichi, Japan [15].

To ensure accurate characterization of adverse effects and evaluation of tolerance, the subjects were asked to keep a patient diary, such as drug compliance and medical conditions, documenting their condition during treatment, with a blister sheet sent to the data center every month.

### Trial end points

Colonoscopy to measure the number, diameter and height of polyps was performed at two time points, before the start and at the end of the trials, as follows. Only one visual field per subject, in which at least four polyps could be detected, was tattooed with ink before the start of the trials. For subjects who had more than one area of multiple polyps, the field from which the best frontal view of the polyps could be obtained was selected. We took several photos using a clamp with a 2-mm scale placed near the tattooed area. Then we measured the diameter and height of the polyps using the scale shown in the photos. Prior to randomization code breaking, changes of polyp were assessed by the Data Evaluation Committee in a blinded manner, comparing endoscopic photographs taken before and after aspirin treatment. The primary end point was set as the presence or absence of an increase in mean diameter of all colorectal polyps observed compared with that before taking aspirin. The secondary end points included change in polyp height, mean diameter and number of polyps, and adverse effects.

### Immunohistochemical staining

Colorectal polyps sampled during colonoscopy were fixed, embedded, and sectioned for immunohistochemical examination with the avidin–biotin complex immunoperoxidase technique, using a polyclonal goat anti-COX-2 antibody (Santa Cruz Biotechnology Inc., Santa Cruz, CA) and a polyclonal goat anti-β-catenin antibody (Santa Cruz Biotechnology) at 100× dilution. As the secondary antibody, biotinylated anti-goat IgG (Vector Laboratories Inc., Burlingame, CA) was employed at 200× dilution. Staining was performed using avidin–biotin reagents (Vectastain ABC reagents; Vector Laboratories), 3,3′-diaminobenzidine, and hydrogen peroxide, and the sections were counterstained with hematoxylin to facilitate orientation. As negative controls, consecutive sections were immunostained without exposure to the primary antibody.

### Statistical analysis

The target sample size was initially set at 100 (50 per group), with the following rationale. To obtain 20% polyp regression in the placebo group and 50% in the aspirin group, 39 subjects were needed for each group to ensure a power of 80% with a two-sided 5% significance level. Considering the possibility of trial dropouts, the target number was increased.

Baseline characteristics were tested by the chi-square test or the Student's *t*-test. “Response rate” was defined as the percentage of subjects with reduced polyp size and number after drug administration among all the subjects of the same group compared with preadministration. Response ratio = aspirin response rate (number of subjects with reduced polyps/total)/placebo response rate (number of subjects with reduced polyps/total). Change in polyp quantitative data was tested by calculating the response ratios and 95% confidence intervals. Adverse event rates of both arms were compared using the chi-squared test. Fisher's exact probability was applied, if needed due to sparse cells in a table. These analyses were also performed in several subgroups. All statistical analyses were intention-to-treat based and performed using PC-SAS (Version 9.1; SAS Inc., Cary, NC) with *P*-values less than 0.05 considered statistically significant.

## Results

### Recruitment of subjects

**Figure 1:**
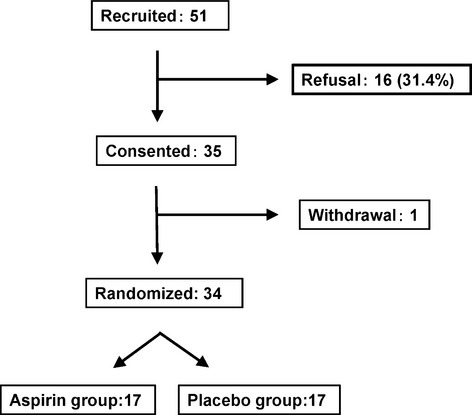
Flowchart for subject recruitment.

A total of only 51 patients could be recruited, of whom 35 (69%) provided informed consent, and took aspirin or placebo tablets. When 10 subjects underwent the end-of-trial colonoscopy, an anastomotic ulcer and severe anemia with decrease to below 3 mg/dL of hemoglobin were observed in one subject receiving aspirin. Consequently, the coresearchers and the Ethics Monitoring Committee decided to cancel further recruitment. Of note, the randomization results were not disclosed until the end of the trial. To conduct strict monitoring during follow-up, we frequently checked the subjects for abdominal pain or symptoms of anemia. We were ready to discontinue trial drug administration and perform blood tests, colonoscopy, and upper esophagogastroduodenoscopy earlier than planned if these conditions developed in the subjects. Fortunately, no subject after the first subject randomized presented with abdominal pain or symptoms of anemia during the trial, and all subjects except for one subject were able to complete trial drug administration as scheduled. The subjects who had already agreed to participate in the trial continued to take the aspirin or placebo with close monitoring of their symptoms (Fig. 1). In addition, one of the subjects who consented to participate in the trial was not included because of involvement in a severe traffic accident before assignment. Thus, 17 subjects each were allocated to the aspirin and placebo groups and completed the trial.

### Characteristics of subjects

The characteristics of the subjects of aspirin and placebo groups are shown in Table 1. There were no statistically significant differences between the two groups regarding sex, age, surgical history, drinking status, smoking status, and *APC* gene mutation.

**Table 1:** Characteristics of subjects in the aspirin and placebo groups

	Aspirin group (*n* = 17)	Placebo group (*n* = 17)
Age (years), mean ± SD	39.7 ± 12.8	36.7 ± 13.9
Body mass index,^1^ mean ± SD	22.4 ± 3.4	21.7 ± 2.1
Treatment period^2^ (days), mean ± SD	250.2 ± 58.0	255.2 ± 57.6
Compliance (%), mean ± SD	83.3 ± 23.8	88.4 ± 10.6
Number (%) of subjects		
Male	8 (47)	9 (53)
Female	9 (53)	8 (47)
Current smoker	4 (24)	4 (24)
Alcohol drinker^3^	7 (41)	5 (29)
Undergone colectomy with IRA	4 (24)	3 (18)
Mean tumor diameter <2 mm	14 (82)	11 (65)
With *APC* gene mutation^4^	14 (82)	12 (71)

SD, standard deviation; IRA, ileorectal anastomosis.

1 Body mass index = weight (kg)/height (m) squared.

2 Data obtained at end of trial.

3 Alcohol drinker: drinks more than once a week.

4 *APC* gene mutation was detected by protein truncation test assay.

### Effect of aspirin on polyps in FAP patients

Subjects in the aspirin group tended to demonstrate greater reduction in the diameter of their colorectal polyps, the primary end point, than the subjects in the placebo group, with a response ratio of 2.33 (95% confidence interval: 0.72–7.55). However, the difference obtained in a response ratio was not statistically significant (Table 2).

**Table 2:** Number of subjects with reduced polyps in aspirin and placebo groups

	Aspirin group (number of subjects with reduced polyps/total)	Placebo group (number of subjects with reduced polyps/total	Response ratio	95% Confidence interval
All subjects	7/17	3/17	2.33	0.72–7.55
Subjects with mean polyp diameter at baseline				
≤2 mm	5/14	0/11	*P *= 0.046^1^	
>2 mm	2/3	3/6	1.33	0.43–4.13
Polyp height	10/17	5/17	2.00	0.87–4.62

1 Fisher's exact probability test.

**Figure 2:**
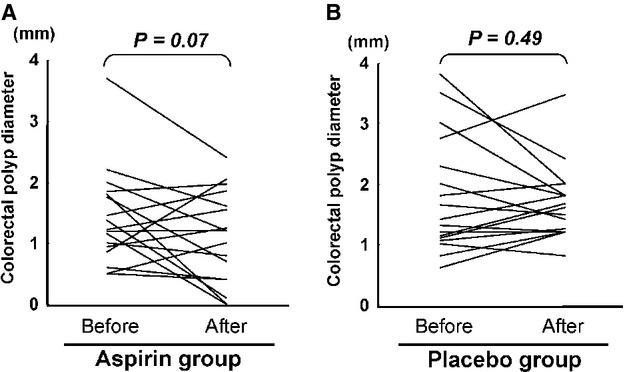
Endoscopic evaluation of polyp size. (A) Changes of mean colorectal polyp diameter in aspirin group. (B) Changes of mean colorectal polyp diameter in placebo group.

Subgroup analysis revealed that number of subjects with a mean baseline polyp diameter of ≤2 mm had a significant reduction in the aspirin group (Table 2). Five of 14 subjects had a significant reduction in the number of polyps in the aspirin group, while no patient had a change in the placebo group (Table 2). Although we should view this small-size study with caution, the diameter of polyps before intervention was almost the same in the aspirin group (1.66 ± 0.61 mm [mean ± SD]) and placebo group (1.78 ± 0.96 mm). After intervention, the diameter of polyps significantly decreased in the aspirin group (1.09 ± 0.75 mm [*P *<**0.05]) compared with that in the placebo group (1.41 ± 0.78 mm) (Fig. 2). Moreover, the number of polyps was smaller in the aspirin group than in the placebo group after intervention. The number of polyps before intervention was almost the same in the aspirin group (2.82 ± 1.54 [mean ± SD]) and placebo group (2.53 ± 1.38). After intervention, the number of polyps significantly decreased in the aspirin group (2.18 ± 1.69 [*P *<**0.05]) compared with that in the placebo group (2.53 ± 1.38). The polyp height tended to exhibit a greater decrease in the aspirin group with a response ratio of 2.0 (Table 2). Thus, the number of subjects with a mean baseline polyp diameter of ≤2 mm, and the diameter and number of polyps after intervention were shown to be the items that achieved significant difference in the subgroup analysis. Moreover, none of the subjects developed colorectal cancer.

### Adverse events of aspirin on FAP patients

There were no serious adverse effects in the placebo group. Of 17 subjects assigned to the aspirin group, three experienced severe adverse effects (18%) (*P *=**0.23, vs. placebo group). These effects included anastomotic ulcer, aphtha in the large intestine, and progression of anemia (3 mg/dL reduction of Hg). All of these subjects were nonsmoking women aged <40 years with high β-catenin staining of their polyps on immunohistochemical examination. A giant anastomotic ulcer was detected at the suture site of the ileal porch in one of the subjects (Fig. S1); therefore, the drug was discontinued after colonoscopy. The ulcer healed as confirmed by endoscopy at 6 months after discontinuation. Aphtha in the large intestine in the second subject was confirmed to have disappeared at the next colonoscopy. Anemia in the third subject improved after cessation of exposure to aspirin. These two subjects had no history of surgery.

## Discussion

In this double-blind, randomized trial, low-dose aspirin enteric-coated tablet administration tended to reduce the size of colorectal polyps in subjects with FAP, as compared with the placebo group. Furthermore, subgroup analysis indicated that the number of subjects with a mean baseline polyp diameter of ≤2 mm had a significant reduction in the aspirin group. Moreover, the diameter and number of polyps significantly decreased, and polyp height tended to decrease. However, we could not draw a definite conclusion from the subgroup analysis as the sample size was too small.

Loss of the *APC* gene and/or dysfunction of APC is known to influence degradation of β-catenin, increasing the levels of β-catenin, and leading to activation of COX-2 through β-catenin/Tcf-4 transcription complexes [12]. It is well established that prostaglandins produced by COX-1 and COX-2 play important roles in colorectal cancer development, and aspirin blocks the activity of both of these enzymes [16]. Moreover, 80% of sporadic colorectal cancers feature *APC* gene somatic mutations, which may appear very early during colorectal carcinogenesis [17]. Thus, the present results indicated that aspirin might act at a relatively early stage of colorectal tumor development through inhibition of prostanoid synthesis.

Recently, Burn et al. reported the results of their CAPP1 study, an international, multicenter, randomized, placebo-controlled trial of aspirin (600 mg/day) and/or resistant starch (30 g/day) for 1–12 years in FAP patients (10–21 years of age) [14]. According to their report, there were no adverse effects due to aspirin, but no reduction of polyps was observed either.

In this clinical trial, we could not achieve the targeted number of subjects because adverse effects occurred during the course of the investigation. All of our subjects who experienced adverse effects were in the aspirin group, and improvement was observed after discontinuation of exposure, suggesting a causative relationship. Adverse effects, such as anastomotic ulcer, aphtha in the large intestine, or progression of anemia was observed in 3 of 17 subjects. We do not have any evidence to explain this high incidence of adverse effects. It is considered that unexpected genetic backgrounds, such as polymorphisms, mutations, and epigenetic changes, might have affected aspirin metabolism. Further examination is required to clarify the reason. Adverse effects due to aspirin in healthy individuals have been reported to occur more frequently in elderly people [18], so that there might be differences between healthy individuals and subjects with FAP in this respect. In the previous clinical trial of sulindac reported by ourselves [4], the similar very high incidence of adverse effects in FAP subjects supported this conclusion. In our previous trial on sulindac, one patient developed a gastric perforation requiring emergency surgery. In this trial, again a serious adverse effect, a giant anastomotic ulcer, was observed. We thought the effect was too severe to continue further recruitment, although gastrointestinal mucosal injury was one of the predictable adverse effects. In future clinical trials of low-dose aspirin enteric-coated tablets in this high-risk group, dose reduction in young women or in those with *APC* gene alteration, or concomitant use of a proton pump inhibitor might be considered.

We would like to emphasize that the low-dose aspirin enteric-coated tablet used in the trial is identical to that widely used for antiplatelet treatment across the world, including Japan. Obvious advantages are the great deal of experience with its long-term use and good safety profile. It was reported that the incidence of adverse effects (including mild ones) was 2.67% [19] according to postmarketing surveillance in Germany conducted by Bayer, or 6.54% according to the survey on Kawasaki disease in Japan by the Ministry of Health, Labour and Welfare [20].

Interestingly, our three subjects with marked symptoms of adverse effects had a relatively large tumor size reduction in common, possibly indicating shared sensitivity for both beneficial and adverse effects. It is necessary to develop a method to identify patients who would benefit from aspirin with a low risk of adverse effects, and to determine the optimal dose. A recently developed technology, as evidenced by a genome-wide association study [21], may help in this respect.

There were several limitations of this trial. First, the sample size was small and second, the evaluation was limited to the tattooed area, without covering the entire colon. However, the reasons we chose these conditions were that it was difficult to objectively evaluate the changes in polyps in the entire colon as it might increase the burden on patients, and that other studies conducted to date also used a similar evaluation method to ours.

In conclusion, the potential for aspirin to reduce colorectal adenoma growth or development in patients with FAP is indicated. However, careful follow-up is needed to avoid and promptly treat adverse effects, with efforts made to identify and verify characteristics of sensitive patients.

## Supporting Information

Figure S1 Giant anastomotic ulcer detected by colonoscopy.
