# Supplementary figures and images for: Preventive effects of low-dose aspirin on colorectal adenoma growth in patients with familial adenomatous polyposis: double-blind, randomized clinical trial
Source: Cancer Med. 2013 Feb 3;2(1):50–6. doi: 10.1002/cam4.46 (PMC3797560; doi:10.1002/cam4.46)

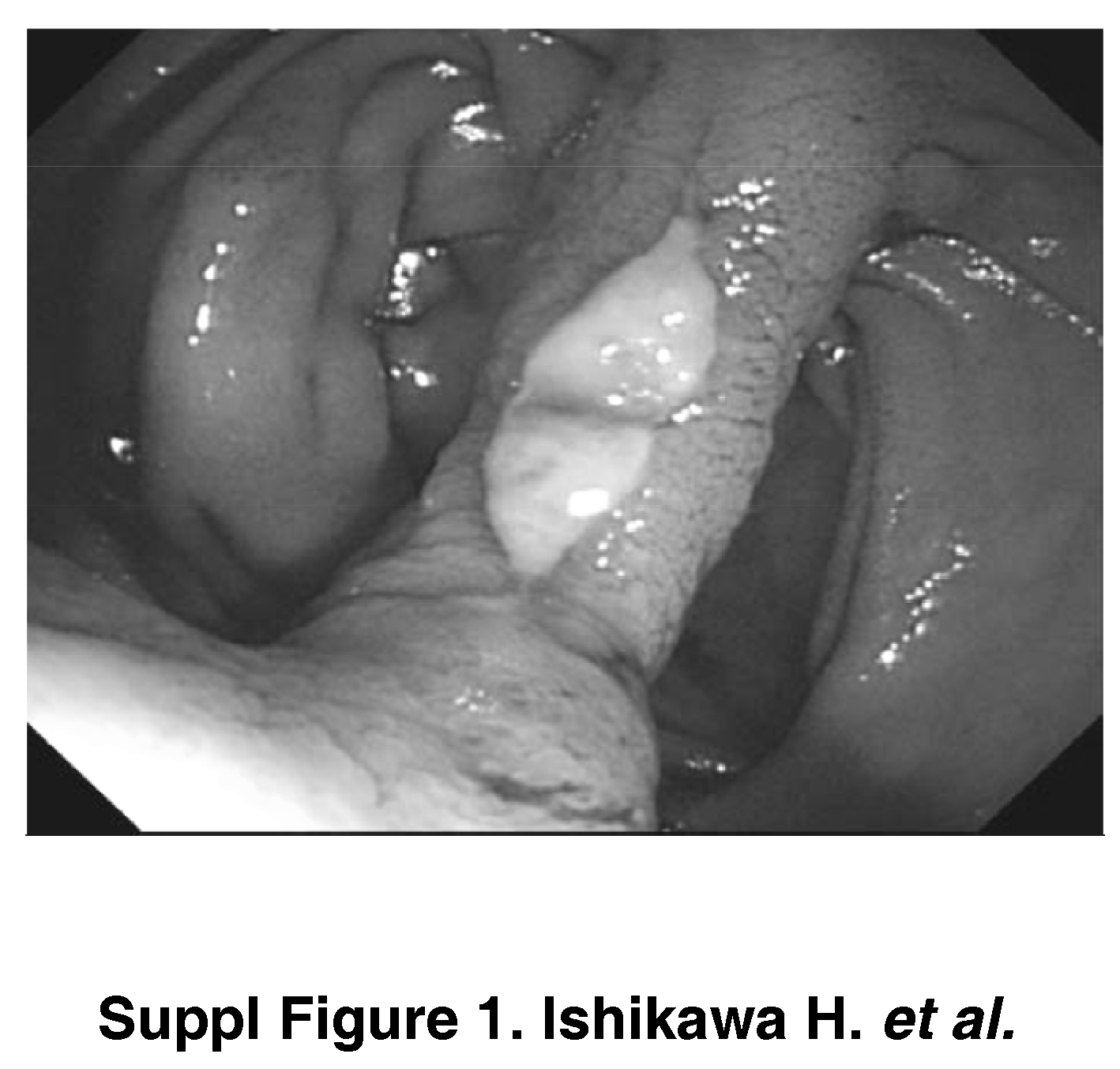

Supplement: Figure S1 — Giant anastomotic ulcer detected by colonoscopy. [file cam0002-0050-SD1.tif]
